# A Retrospective Study of Macropod Progressive Periodontal Disease (“Lumpy Jaw”) in Captive Macropods across Australia and Europe: Using Data from the Past to Inform Future Macropod Management

**DOI:** 10.3390/ani10111954

**Published:** 2020-10-23

**Authors:** Jessica Rendle, Bethany Jackson, Stephen Vander Hoorn, Lian Yeap, Kristin Warren, Rebecca Donaldson, Samantha J. Ward, Larry Vogelnest, David McLelland, Michael Lynch, Simone Vitali, Ghislaine Sayers, Fabia Wyss, Darren Webster, Ross Snipp, Rebecca Vaughan-Higgins

**Affiliations:** 1 Conservation Medicine, College of Science Health, Education and Engineering, Murdoch University, Perth 6150, Australia; b.jackson@murdoch.edu.au (B.J.); l.yeap@murdoch.edu.au (L.Y.); k.warren@murdoch.edu.au (K.W.); r.donaldson@murdoch.edu.au (R.D.); r.vaughan-higgins@murdoch.edu.au (R.V.-H.); 2 Twycross Zoo, Atherstone, Warwickshire CV9 3PX, UK; samantha.ward@ntu.ac.uk; 3 Brackenhurst Campus, School of Animal, Rural and Environmental Sciences, Nottingham Trent University, Southwell, Nottinghamshire NG25 0QF, UK; samantha.ward@ntu.ac.uk; 4 School of Population and Global Health, University of Western Australia, Perth 6009, Australia; stevevdh@hotmail.com; 5 Taronga Conservation Society, Mosman 2088, Australia; LVogelnest@zoo.nsw.gov.au; 6 Adelaide Zoo, Adelaide 5000, Australia; dmclelland@zoossa.com.au; 7 Melbourne Zoo, Parkville 3052, Australia; Mlynch@zoo.org.au; 8 Perth Zoo, South Perth 6151, Australia; simone.vitali@dbca.wa.gov.au; 9 Paington Zoo, Painton, Devon TQ4 7EU, UK; Ghislaine.Sayers@paigntonzoo.org.uk; 10 Basel Zoo, 4054 Basel, Switzerland; fabia.wyss@zoobasel.ch; 11 Blackpool Zoo, Blackpool, Lancashire FY3 8PP, UK; Darren.Webster@blackpoolzoo.org.uk; 12 Flamingo Land, Malton, Yorkshire YO17 6UX, UK; ross.snipp@flamingoland.co.uk

**Keywords:** kangaroo, wallaby, epidemiology, zoo, dental disease, animal welfare

## Abstract

**Simple Summary:**

Macropod Progressive Periodontal Disease (MPPD), or ‘lumpy jaw’, is an often-fatal dental disease commonly reported in captive kangaroos and wallabies (macropods) worldwide. The disease is difficult to treat successfully, resulting in high recurrence and mortality rates. The aim of this study was to determine animal and environmental factors that may increase the risk of developing MPPD. We conducted a multi-institution study of MPPD in macropods in zoos in Australia, and compared data with those in European zoos, where macropods are popular exhibit animals. This study reports risk factors for the development of disease including region, age, sex and particular stressors, such as transport between enclosures and between zoos. This information contributes to the understanding of disease development and advances the evidence base for preventive management strategies. We recommend protocols to reduce or prevent outbreaks of MPPD in zoos, thus decreasing morbidity and mortality rates of this challenging disease. The implementation of these recommendations will benefit the welfare and health of captive macropods worldwide.

**Abstract:**

Macropod Progressive Periodontal Disease (MPPD) is a well-recognised disease that causes high morbidity and mortality in captive macropods worldwide. Epidemiological data on MMPD are limited, although multiple risk factors associated with a captive environment appear to contribute to the development of clinical disease. The identification of risk factors associated with MPPD would assist with the development of preventive management strategies, potentially reducing mortality. Veterinary and husbandry records from eight institutions across Australia and Europe were analysed in a retrospective cohort study (1995 to 2016), examining risk factors for the development of MPPD. A review of records for 2759 macropods found incidence rates (IR) and risk of infection differed between geographic regions and individual institutions. The risk of developing MPPD increased with age, particularly for macropods >10 years (Australia Incidence Rate Ratio (IRR) 7.63, *p* < 0.001; Europe IRR 7.38, *p* < 0.001). Prognosis was typically poor, with 62.5% mortality reported for Australian and European regions combined. Practical recommendations to reduce disease risk have been developed, which will assist zoos in providing optimal long-term health management for captive macropods and, subsequently, have a positive impact on both the welfare and conservation of macropods housed in zoos globally.

## 1. Introduction

Macropod Progressive Periodontal Disease (MPPD), colloquially referred to as ‘lumpy jaw’, is a disease that is reported in macropods in zoological collections worldwide and is considered a leading cause of death of kangaroos and wallabies [1,2,3,4]; however, epidemiological data examining the level of risk and factors associated with the development of clinical disease are limited. The frequent occurrence of MPPD in zoo macropods, compared to their wild counterparts, where little to no evidence of the disease is reported [2,5], suggests an association between the disease and aspects of the captive environment [2,4,6,7,8]. Therefore, an epidemiological study identifying the factors associated with the occurrence of MPPD would aid in the development of preventive strategies, ultimately reducing morbidity and mortality rates of this challenging disease.

MPPD is a disease of multifactorial aetiology, and the potential triggers for the disease are proposed to originate from both environmental and animal-centred sources, including the presence of infectious agents [1,2,9]. Several bacterial species have been associated with MPPD, although *Fusobacterium necrophorum* is frequently named as a causative agent associated with the disease [1,2,9]. The species has been cultured from the oral cavities of macropods affected with the disease, and has also been reported to survive under certain conditions in the environment [1]. Other reported risk factors have been reported to include feeding strategies, such as diet and methods of dietary presentation [6,10], stocking densities, enclosure hygiene [6,11,12], cold climates [3,13,14] and potential sources of stress such as visitor presence and proximity, confinement, transport and climatic conditions including freezing temperatures, drought and seasonal variation in temperatures [1,2,12,15,16,17,18]. In captivity, the location of the host institution and institutional management strategies predominantly control these factors. Precursors for MPPD may also originate from the host, with the presence of periodontal disease and the processes of tooth eruption and molar progression cited as potential contributing factors to the disease [2,8,19]. The rates at which macropod teeth erupt, and at which molar progression occurs, vary between genera and species, and correlate with age [19,20,21,22]. Therefore, the association between host-related dental development and the incidence of MPPD potentially varies between macropod genera and age. In some macropod species, sexual dimorphism in dentition and body mass is reported [22,23]; however, a sex bias in the development of MPPD is yet to be identified. While these host-related risks for MPPD may be challenging to control, exposure to environmental triggers for the disease are predominantly manageable by the institution. To date, the effects that these differences have on the incidence of MPPD have not been substantially investigated.

Although a recent study reported an absence of MPPD in wild macropods [5], the disease in captive macropods has been reported across genera and countries; however, there is variability in the reporting of the disease between genera, species [2], and between reporting institutions [2,3]. Studies of MPPD, to date, have been limited by the composition of the study population, the application of crude disease frequency measures (prevalence across species and prolonged time frames), and the inherent complications of confounding; the wide variety of environmental and host influences on this disease undermine the ability to derive comparable study populations in captivity. The multifactorial and chronic nature of the disease further complicates research design, as experimental infection studies cannot be applied, and prospective cohort studies ideally require fixed management of study populations over extended time frames, conditions that are not easily achieved in zoos due to a range of factors. The majority of reports have used biased study populations, where prevalence (P) is reported either from deceased individuals (e.g., 2,21), or by comparing deaths from MPPD to all other causes [13]. These studies do not include data from macropods that were successfully treated and survived a case of MPPD, nor the entire population at risk; therefore, these results do not reflect the full extent of the disease in ‘extant’ captive populations. Importantly, most studies describe period prevalence (cases of MPPD over time periods that may extend to years), rather than an incidence rate (IR) that takes into account the animal time at risk and provides for dynamic populations where animals enter and leave the study population throughout the period of reporting [24]. Whilst prevalence reflects the disease burden in a given population over time, the incidence rate (animals developing disease/total animal time at risk) and incidence rate ratio (IRR) (comparison between incidence rates for different risk factors) are better suited to investigating disease aetiology and risk factors [25]. Therefore, incidence rate and incidence risk ratio are the more appropriate measurements to apply to captive study populations of macropods with MPPD. This is particularly the case given that MPPD prevalence will likely be inflated by the chronicity of disease if datasets cover extended time periods.

The reported frequency of MPPD in captive macropod populations worldwide both compels and provides for epidemiological investigations of the disease. Using zoo animal records, the aims of this research were to: (1) determine the regional prevalence of MPPD in captive macropods housed across two regions where macropods are popular exhibits: Australia and Europe; and (2) to systematically examine temporal trends in the incidence of the disease (the incidence rate (IR)) and explore animal and environmental risk factors for the development of MPPD (incidence rate ratios (IRR)).

## 2. Methods

Ethics approval for this study was given by the Murdoch University Animal Ethics Committee (Permit Number R2754/15) and Human Ethics Committee (Permit Number 2015/182). Participating institutions provided their own approval where necessary (Zoo A2: ZV16005; Zoo A3: 2015-6; Zoo A4: R16D217).

### 2.1. Case Definition

**Figure 1:**
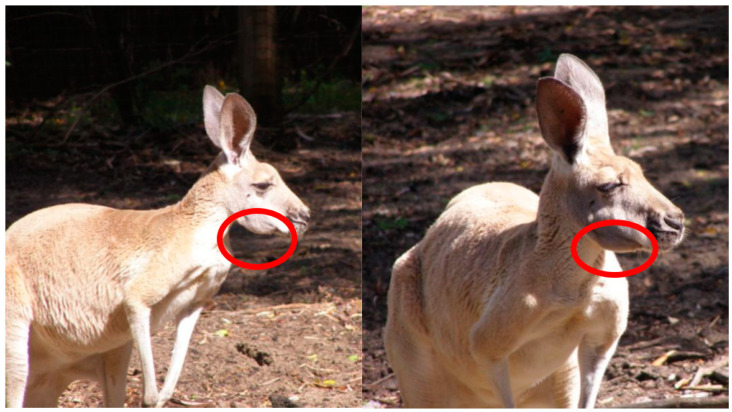
Swelling of the mandibular region (encircled) associated with clinical signs of Macropod Progressive Periodontal Disease (MPPD) in a red kangaroo (*Macropus rufus*). (Images courtesy of Perth Zoo).

**Figure 2:**
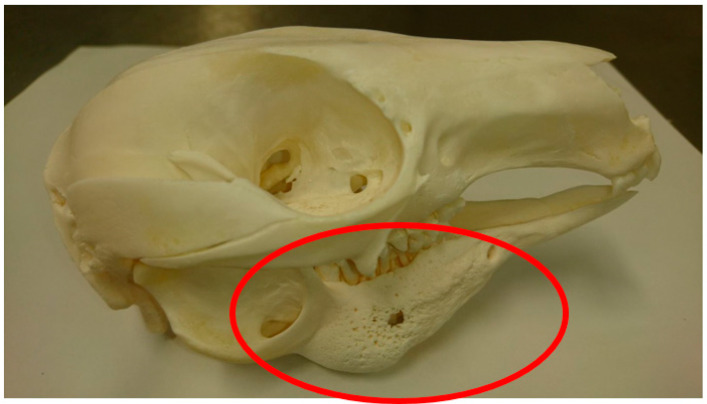
Osteoproliferation consistent with osteomyelitis in the mandibular bone of a western grey kangaroo (*Macropus fuliginosus*). (Image courtesy of P. Dobbs, Twycross Zoo).

The challenge of capturing true cases of MPPD is that clinical MPPD is considered to be a continuum of oral disease [26]. MPPD progresses from gingivitis, in the early stages, through to periodontitis, advancing further to involve the bones of the mandible and/or maxilla (Figure 1), whereby necrotising osteomyelitis of the jaw (Figure 2), with periosteal new bone formation, is observed [26]. As the early stages of MPPD are conditions in their own right, the development of a clear case definition was required, to differentiate MPPD from other oral diseases. A case definition for MPPD was developed using reported descriptors of the disease and tools commonly used in the diagnosis of MPPD. Our definition differs from the recent proposal by McLelland (2019), in that ours focuses on the final stages of disease, rather than the inclusion of the earlier progressive stages, which may or may not progress to osteomyelitis. The definition was circulated to a focus group of 16 zoo professionals and veterinarians for feedback on sensitivity, specificity and clarity. Feedback was used to develop the final case definition, which was used to capture mid to later stages of the disease reported in clinical records. In addition to the use of terminology previously used to describe cases of the disease, such as ‘lumpy jaw’, in this study, we considered a case of MPPD to be:

“Proliferative bony change of the maxilla/mandible or soft tissue inflammation (lumps), and/or radiographic/visual evidence of osteomyelitis/osteolysis; accompanied by dental disease. There may or may not be demonstrable bacterial involvement through microbial culture.”

### 2.2. Selection of Study Institutions

The Zoological Information Management System (ZIMS) was used to generate Species Holding Reports (SHR) for genera under the Family ‘Macropodidae’ (genus: *Dendrolagus*, *Dorcopsis*, *Lagorchestes*, *Onychogalea*, *Macropus* (*including *Notomacropus* and *Osphranter*), *Petrogale*, *Thylogale*, *Setonix* and *Wallabia*) for two regions—Australia and Europe. Institutions were selected primarily on their current holding of the western grey kangaroo (*Macropus fuliginosus*), the focal species for another aspect of the larger research project. Institutions were also selected based on knowledge of a previous history of lumpy jaw from personal communication, or literature-based evidence, and on existing established links with individuals involved in this study. Eight zoos were selected: four from Australia, and four from Europe. Institutions were anonymised: institutions in the Australian region were identified using the prefix A, with the four individual institutions referred to as Zoos A1–A4, respectively, and European institutions had the prefix E, and were referred to as Zoos E1–E4. For the purposes of this study, macropods that have since been re-classified were included in the data for *Macropus*.

### 2.3. Extraction of Electronic Records

#### 2.3.1. Taxon Reports

Institutions used a variety of methods for the storage of biological and clinical data; therefore, we collected all electronic and paper-based records available, and each institution was visited to personally collect the records required for this research. To identify macropods housed at each institution during the study period, we used institutional access to ZIMS and made specific selections to capture only the ‘Local’ animals that were part of the ‘Main Institution Animal Collection’, as well as those that were ‘Owned and On Site’ between our selected dates (from 1 January 1995 to the date of data collection). Each taxonomic genus was selected in turn, and reports generated information which held the individual’s Global Accession Number (GAN), a unique identifier that was used for the generation of all further ZIMS reports required.

#### 2.3.2. Medical Records (ZIMS and Filemaker)

Medical records provided information on the diagnosis of MPPD, date of diagnosis, and an outcome. ZIMS Medical (Species360, Minneapolis, MN, USA. Version 2.2, then Version 2.3 after 25 January 2016) was used for the extraction of clinical records and a selection was made for ‘Full Medical History’. The GAN for each individual animal was entered, and a selection was made for all record types, and the report was generated. For each animal for which a medical history was available, the report was downloaded and saved in pdf format for later review.

One zoo recorded their clinical data using FileMaker^®^ Pro software (Filemaker, Inc., Santa Clara, CA, USA. Version Pro/Server 9*), where all clinical and associated information relevant to the individual macropods were stored. Records were located by searching for species housed at the zoo, for example ‘*Graues Riesenkänguru*’ (western grey kangaroo). Google^®^ Translate (Google^®^ Inc., Mountain View, CA, USA) was used to translate all information from German into English, and terminology that could not be translated through online means were confirmed by conversing with the veterinary team at the zoo.

#### 2.3.3. Specimen Reports

Specimen Reports (SR) contained data relating to life history, current location (if still alive and present in the zoo), sex, birth, inter- and intra-zoo transfers and some death information. Reports were generated using the GAN for each individual macropod, and the date range was selected from 1 January 1800 to the date of data collection, as this enabled an animal’s full life history to be found. Each report was downloaded and saved for later review.

#### 2.3.4. Notes and Observations

The ‘Notes and Observations’ sections in ZIMS were generally used by keepers to diarise information regarding changes in animal behaviour, diet or general husbandry. However, this section was also heavily used for the reporting of health issues and medical treatments at some institutions. The Notes and Observations were located by first doing a simple ‘Animal’ search using the individual’s GAN where the data relating to a particular animal could then be downloaded.

### 2.4. Extraction of Paper-Based Records

All the zoos included in this study held some paper-based records which included clinical records, necropsy reports, and keeper records. Paper records were scanned using a handheld scanner (Epson^®^ Australia Pty Ltd., North Ryde, Australia) and saved as pdf copies for later review.

### 2.5. Inter- and Intra-Zoo Transfers

Inter-zoo transfers were recorded on the Specimen Reports as a transfer between ‘physical holders’. Any transfer between physical holders was counted as one inter-zoo transfer up to the point of entry into its current location. Macropods that entered a zoo on multiple occasions had their total number of transfers calculated. Animals that were brought in from the wild, and remained in captivity (given a GAN), were reported as one transfer, unless further information about them was known. A reported macropod escape, and subsequent return to an enclosure, was not classed as a transfer. All animals with an unrecorded history were reported as ‘unknown’.

A combination of institutions’ Specimen Reports, Notes and Observations, and Enclosure Reports were used to identify the number of intra-zoo transfers each animal experienced. All transfers between ‘on exhibit’, ‘off exhibit’ and ‘hospital’ transfers were counted. Records also indicated that transfers occurred between hospital enclosures, and these were also included in the number of intra-zoo transfers. Not all institutions recorded the intra-zoo transfers of their animals, in which case these individuals were recorded as ‘unknown’. Intra-zoo transfers recorded on ZIMS were for the animal’s lifespan at the zoo; therefore, the figures provided for total intra-zoo transfers reflect the number of enclosure moves that each animal experienced for their total lifespan at the institution, rather than solely for the study period.

### 2.6. Selection of Study Population

All macropods recorded as being housed at participating institutions during the study period were included in the analyses. However, the literature suggests that MPPD is not detected in pouch young (PY) [3], and arguably, PY would not be exposed to all of the same risk factors for MPPD as adults. The youngest reported age of macropods to permanently exit the pouch is 119 days, or 0.3 years (bridled nail-tail wallaby *Onychogalea fraenata*) [27]; therefore, we only included macropods that were >0.2 years (73 days) at the time of date collection, to remove the influence of outliers. The small sample prevented analyses at the species level; therefore, macropod populations were analysed at the genus level.

### 2.7. Statistical Analyses

#### 2.7.1. Prevalence and Odds Ratio Calculations

Data were entered into Microsoft^®^ Excel 2016 (Microsoft^®^ Corporation, Washington, DC, USA) for initial exploration. Prevalence was defined as the proportion of macropods within the population identified in the records as being affected by MPPD at some point during the retrospective period of 1 January 1995 to 31 December 2015. Prevalence (P) and confidence intervals (CI) were calculated using the exact binomial method [28] and the calculation of odds ratios (OR) were performed using Epitools [29]. Chi-square tests for measures of significance were used when all categories were greater than five, and two-tailed Fisher’s exact tests when any one category was less than five. Measures of difference for geographical region were assessed using the chi-square test as calculated in Epitools [29] with the measure of significance taken at *p* ≤ 0.05.

#### 2.7.2. Incidence Rate Calculations

Monitoring time was defined as being from the initial date of arrival into an institution (for example, from birth or transfer in) or data were trimmed from 1 January 1995 (whichever was later), until the date of the first incidence of MPPD, death, or an animal being lost to follow up (whichever occurred first). The follow-up dates for some individuals went as far as 2016 (until 28 November 2016, the last date when data were extracted from ZIMS). Animals with no date of arrival or departure were removed from IR and IRR analyses. Incidence rate ratios were calculated for animals grouped by calendar period (1995–1999, 2000–2004, 2005–2009, 2010–2016), age group (<1 year, 1–4 years, 5–9 years, ≥10 years), sex (male, female), genus, and institution. Incidence rates were calculated by dividing the number of cases by the monitoring time at risk and presented per 100 animal years. Ninety-five percent confidence intervals were derived using the exact Poisson method.

Analyses involving between-group comparisons were conducted using Poisson regression modelling, with an offset term set to the number of monitoring years. Adjusted IRRs were calculated by setting a reference category for each factor and comparing each of the remaining levels to the corresponding reference category, i.e., the reference category was ‘1995–1999′ for calendar period; ‘<1 year’ for age group, and ‘females’ for sex. Comparisons between institutions were conducted using the institution with the lowest unadjusted incidence rate for each region separately. All models included calendar period, age group, sex, and zoo as separate terms to facilitate the estimation of an adjusted IRR. The IR and IRR presented were calculated in R version 3.3.3 [30].

## 3. Results

Examination of 6178 animal records revealed that 2759 macropods were housed across the eight institutions between 1 January 1995 and 28 November 2016. The population considered at risk (>0.2 years) was comprised of 2054 macropods: 1620 in Australia and 434 in Europe, as summarised in Table 1.

### 3.1. Risk Factors

#### 3.1.1. Region

Geographic region was found not to be a significant factor in the development of MPPD (*p* = 0.45; Australia P = 13.8%, 95% CI: 12.2–15.6; Europe P = 12.2%, 95% CI: 9.3–15.7). The IR for MPPD in the Australian region, of 5.7 cases/100 animal years (95% CI: 5.0–6.6), did not significantly differ from the European region at 4.9 cases/100 animal years (95% CI: 3.6–6.5).

#### 3.1.2. Sex

Macropod sex was a significant risk factor for developing MPPD within Europe (Table 2 and Table 3) whereby males in European institutions were two times more at risk of developing the disease than females (IRR 2.02, 95% CI: 1.08–3.83, *p* = 0.03). Macropod sex was not found to be a significant factor for the Australian region (IRR 1.08 cases/100 animal years, 95% CI: 0.80–1.44, *p* = 0.611).

#### 3.1.3. Age

The age at which MPPD was first detected for macropods within the study ranged between 0.3 years and 18.9 years. The mean (±SD) age of onset in this study population was similar in both regions (Australia 5.6 ± 3.6 years; Europe 6.0 ± 5.1 years), although variation was noted between species. The likelihood of developing MPPD increased with age (Table 2), with macropods aged over 10+ years significantly more at risk of developing disease for both Australia (IRR 7.63, 95% CI: 4.06–15.20, *p* = < 0.001), and Europe (IRR 7.38, 95% CI: 2.50–24.85, *p* = < 0.001).

#### 3.1.4. Study Period

The incidence of MPPD remained relatively stable in the Australian region throughout the study period, although a slight reduction in incidence was observed in later years (2010–2016) (Table 2). However, the risk of developing MPPD was not significantly different between any two of the 5-year periods examined (*p* > 0.05) (Table 3). Yet the last 10 years (2005–2016) saw a significant increase in the incidence of MPPD in the European region (Table 2). In recent years (2010–2016), macropods in the European region were nearly seven times more likely to develop MPPD than when recording began in 1995 (IRR 6.94, 95% CI: 1.96–44.18; *p* = 0.010), and were at greatest risk during 2005–2009 (IRR 7.39, 95% CI: 2.06–47.21, *p* = 0.008) (Table 3).

#### 3.1.5. Institution

Institutional IRs indicated that Zoo A3 had the lowest incidence of MPPD of the four Australian institutions throughout the study period, with only 16 cases of MPPD reported between 1995 and 2016 (IR 2.4 cases/100 animal years, 95% CI: 1.4–3.9) (Table 2). The modified Poisson regression model estimated the risk of developing MPPD to be significantly greater for macropods housed at the four Australian institutions, when compared to Zoo A3 (Zoo A1: *p* < 0.001; Zoo A2: *p* < 0.001; Zoo A4: *p* = 0.013) (Table 3). In the European region, the lowest IR was observed at Zoo E4 (IR 3.6 cases/100 animal years, 95% CI: 1.4–7.3) (Table 2); however, the risk of developing MPPD was not statistically different between the European institutions (*p* > 0.05) (Table 3).

#### 3.1.6. Genus

**Figure 3:**
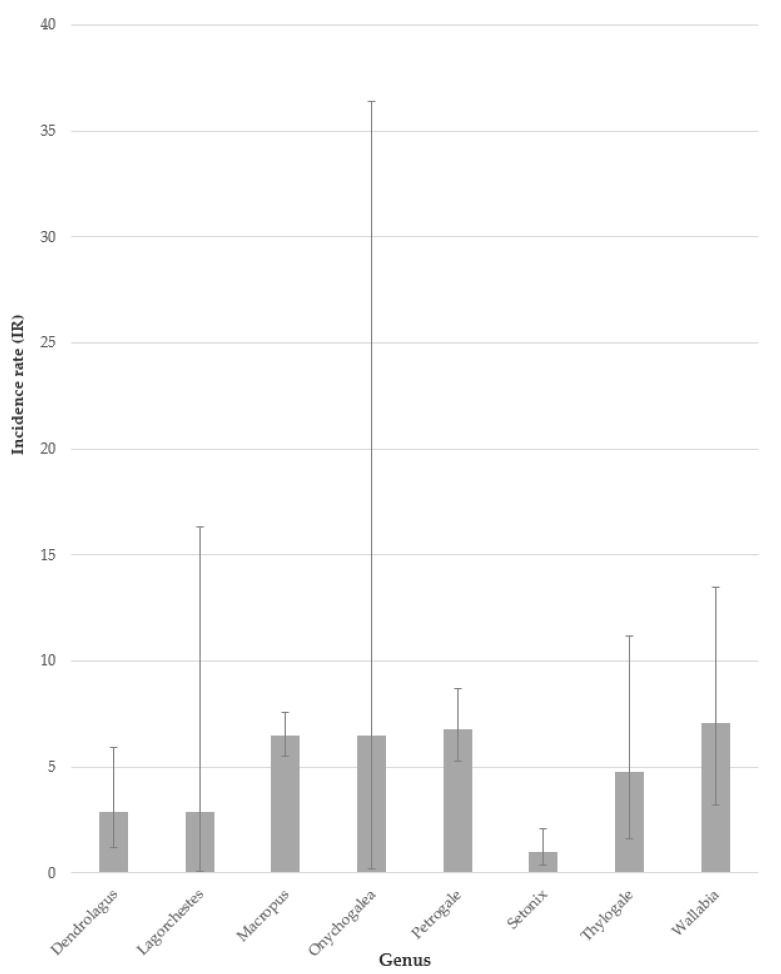
Incidence rates (cases/100 animal years) and 95% CI for MPPD by genus for macropods housed at eight zoological institutions across Australia and Europe between 1 January 1995 and 28 November 2016.

MPPD was identified in all genera within the Macropodidae family, including *Dorcopsis*, although in this genus, disease was noted outside of the study period (cases identified in the records pre-1 January 1995 or post-28 November 2016). The combined regional data determined the greatest IR was in *Wallabia* (IR 7.1 cases/100 animal years, 95% CI: 3.2–13.5), and the IRs for *Wallabia*, *Petrogale* and *Macropus* were all significantly greater than the IR reported for *Setonix* (IR 1.0 cases/100 animal years, 95% CI: 0.4–2.1) (Figure 3).

### 3.2. Inter-Zoo Transfers

Macropods housed in the Australian region experienced up to seven inter-zoo transfers per individual, during the study period. The odds of developing MPPD in the Australian region increased significantly as the number of inter-zoo transfers increased (Table 4). Macropods that experienced only one inter-zoo transfer were 1.7 times more at risk of developing MPPD than macropods that had no inter-zoo transfers (OR = 1.69, 95% CI: 1.23–2.32, *p* = < 0.001). This trend increased further to nearly 44 times the risk for those that had the greatest number of inter-zoo transfers (OR = 43.60, 95% CI: 2.08–915.6, *p* = < 0.01). Macropods in the European region experienced up to three inter-zoo transfers per individual during the study period; however, there were no significant relationships found between the number of inter-zoo transfers and the risk of developing MPPD (Table 4).

### 3.3. Intra-Zoo Transfers

The greatest number of intra-zoo transfers recorded for any individual macropod was 37 transfers per macropod for the Australian region. The odds of developing MPPD increased significantly as the number of intra-zoo transfers increased (Table 5), with macropods that experienced two intra-zoo transfers having two and half times the risk of developing MPPD than macropods with no intra-zoo transfers (OR = 2.68, 95% CI: 1.36–5.30, *p =* 0.003). This trend increased further to over 16 times the risk for those that had the greatest number of transfers (OR = 16.18, 95% CI: 8.73–29.98, *p* ≤ 0.0001). Macropods housed in the European region experienced up to seven intra-zoo transfers (Table 5). There was no significant association found between the risk of developing MPPD and the number of intra-zoo transfers for macropods housed in the European region.

### 3.4. Outcome (First Incidence of Disease)

**Figure 4:**
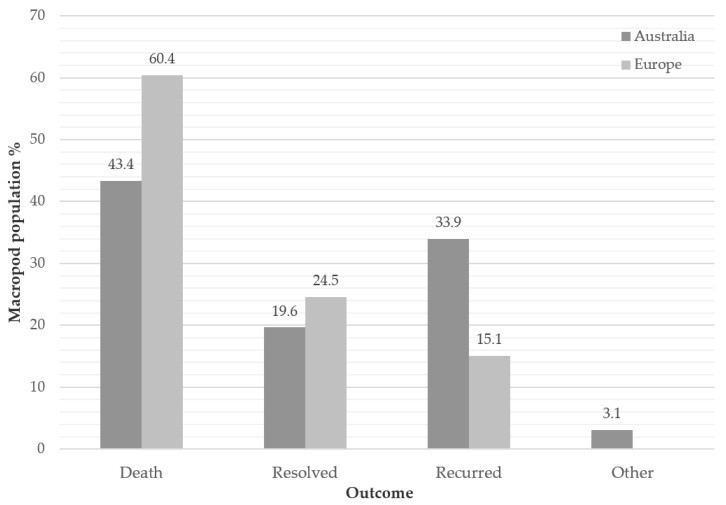
Outcome of the initial case of MPPD reported in zoo records for macropods housed at eight zoological institutions across Australia and Europe between 1 January 1995 and 28 November 2016. In “Recurred” cases, 53.9% (*n* = 76) of Australian cases and 42.9% (*n* = 7) of European cases eventually died. ‘Other’ represents macropods lost to follow up.

The overall mortality rate for initial cases of MPPD was 46.6% for both regions combined. Recurrence was common, to a greater extent in Australia, with more than one third of Australian-housed macropods that experienced a first case of MPPD experiencing at least a second occurrence of the disease (33.9%) (Figure 4). A large proportion of recurrent cases eventually succumbed to the disease (Australia 53.9%; Europe 42.9%) and, across both regions combined, 62.5% of macropods eventually died of the disease (Australia 61.6%; Europe 66.0%).

## 4. Discussion

This is the first retrospective epidemiological study of MPPD in captive macropods across multiple institutions in Australia and Europe, revealing a similar prevalence of the disease within each region from January 1995 to November 2016. However, the risk (IR) of developing MPPD during a macropod’s lifetime in captivity significantly increased across the European region in more recent years (2010–2016), whilst the institutions in the Australian region remained comparatively static for the study period. Risk factor analyses also highlighted regional differences for the significance of sex, time period, and inter- and intra-zoo transfers, which may reflect regionally derived institution-specific management practices. Overall, Australia reported a higher rate of disease recurrence in individuals that survived the initial diagnosis; however, the outcome of MPPD was poor regardless of the region in which macropods were housed.

Retrospective studies, including those that make use of zoo records, are an indispensable tool in the identification of disease patterns [3,31,32,33], and can also be used to evaluate the efficacy of interventions, such as modifications to husbandry practices, aimed at reducing the incidence of a disease [34,35]. Studies such as this provide important information from which zoological institutions can make management decisions that may reduce the risk of MPPD and help to conserve the health and welfare of macropods in captivity. However, retrospective studies can only be successful if records are accurate and complete, particularly for multifactorial disease entities such as MPPD that have case definitions with multiple criteria, which may change over time.

The case definition developed for this study focussed on bony pathology and soft tissue swelling associated with dental disease. This differs from the broader case definition proposed by McLelland (2019) which encompassed pathologies considered to be earlier stages of disease: progressive inflammatory and necrotising polymicrobial disease associated with predominantly anaerobic opportunistic bacterial infection of the soft tissue and bony structures supporting the teeth, including gingivitis, periodontitis and mandibular/maxillary osteomyelitis. In the following discussion of our results, MPPD is defined by the case definition developed for this study. This narrower case definition captured mid to late stages of this progressive disease, and early stages of MPPD may have been missed or misdiagnosed. This may have resulted in an underrepresentation of the extent to which MPPD affects captive macropods across both regions.

### 4.1. Regional Prevalence and Incidence Rates

Crude prevalence results across the study period indicate that the burden of MPPD was similar in captive macropod populations irrespective of the geographic region where they were located; findings that contrast with previous studies suggesting colder climates, specifically, may be a risk factor [3,13,14]. Whilst prevalence results for the disease have been reported elsewhere [1,2,3,5,36,37], these reports were often based on institutional or species-specific data and therefore are less comparable to our regional results. In one example, prevalence reported by Vogelnest and Portas [2] (13.4%), was based on institutional necropsy reports across all species, and although it compares to the 13.8% we reported for the Australian region, again across species, it only reflects the presence of disease at the point of death. Although prevalence is an important measurement of the presence of disease in a population, it does not capture the risk of developing the disease over a lifetime. Importantly, the data provided by zoological institutions, where entire cohorts can be followed, and thus animal time at risk is captured, lends itself to the measure of IRs and IRRs, which more appropriately capture the level of disease and risk factors associated with the occurrence of disease for dynamic populations [24]. Whilst MPPD is reported to be one of the most frequently observed diseases in captive macropods [2,6,8,10,38,39], the IRs calculated during this study were seemingly low based on our expectations. For both Europe (4.9 cases/100 animal years) and Australia (5.7 cases/100 animal years), the IR’s did not differ significantly between the regions. A review of the same dataset for other diseases of macropods and derivation of their IRs would provide the necessary perspective to determine which diseases are in fact most common for captive macropods in these regions. Furthermore, we recommend that future studies determine the IR of MPPD by species, institution and/or region, to compare these to the results presented here.

### 4.2. Risk Factor Analysis

#### 4.2.1. Sex

Contrary to previous reports [2], in our study, the incidence of MPPD differed significantly between sexes, with male macropods more than twice as likely to develop the disease than females in Europe. This may reflect differences in the management of male macropods (such as population structure and sex ratios), resulting in a sex-specific inability to adapt to the captive environment [17,40]. The provision of a captive environment that reflects the natural habitat for the species, as well as the population structure and dynamics specific to the species, is a priority for zoos and underpins individual and population health [41,42]. Same-sex grouping is a method used for population control in zoos [42,43]; however, it does not reflect normal mob structure and may affect macropod behaviour, especially in the more gregarious species commonly led by a dominant male [44,45]. Reduced access to females, and/or an increase in male competitors may be stressful for males and increase agonistic behaviours [44,46,47,48] risking immunosuppression which is a proposed risk factor for MPPD [4,49]. The overall population ratio of males to females in the Australian region was close to 2:3 (643:908) [50], a ratio more akin to that of sex ratios reported in wild mobs, where the ratio is approximately 1:5 (red kangaroo) [45]. However, the overall ratio of males to females in the European region was almost 1:1 (207:209) [50], and if this ratio was representative of the proportions present at the zoo level, and in individual enclosures, this would reflect a sex ratio not present in wild populations.

In addition to physical and behavioural differences between the sexes [8], the agile wallaby (*Notomacropus agilis*) and red kangaroo (*Osphranter rufus*) also show sexual dimorphism in dental development [22,45,51], with males acquiring teeth earlier than females of the same species. Given molar progression is a hypothesised risk factor for MPPD, and early development of teeth would increase the animal time at risk for an individual, there may be a sex-bias in dental development that drives the increased lifetime risk for males. Further investigation into this area should be encouraged. Another consideration is the potential relationship between macropod sex and the diet. Sexual dimorphism in body mass and size-related metabolic needs can result in differences in the diet [23] and selective feeding [52,53,54]. In captivity, where an artificial diet is often presented, selective feeding of preferred food items may affect dental health or development. The regional bias observed in this study may therefore be associated with the sex of the macropod influencing the diet selection, and potentially with regional differences in diet presentation.

#### 4.2.2. Age

The risk of MPPD increased significantly with age, with macropods >10 years of age more than seven times as likely to develop the disease within both regions. This result was unsurprising for a number of reasons, including the relationship between molar progression and dental disease (an age-related condition and also a precursor for MPPD) with the development of MPPD [9,21], and the chronicity of disease with detections often occurring in the later stages. In addition, there is reduced likelihood of detection of MPPD in juveniles (<1 years old), due to the challenges involved in undertaking observations of the oral cavity in smaller individuals. Despite the above, we detected five cases of MPPD in <1 year old macropods; which alongside the finding of 15 juveniles with the disease [3], suggests that younger individuals should not be exempt from routine examinations for this disease. The mean age for developing MPPD was similar across both regions and multiple species (Australia 5.6 ± 3.6 years; Europe 6.0 ± 5.1 years). These mean ages differ to the findings of other researchers [3], who reported a lower mean age of onset at 3.1 ± 2.1 years. However, the studies undertaken by other researchers and ours are both confounded by species, which have differing lifespans and dental development. Thus, the use of a relative age of onset (proportional to life expectancy), or grouping by developmental stages may be more useful. Furthermore, given the large number of inter-zoo transfers observed in this research, cases of MPPD may have been missed due to occurrence outside of our study period, or at another institution not examined in this study. Cases of MPPD not captured in this study may affect the ‘age of onset’, as well as the incidence and prevalence calculated in our study. Incomplete records, especially during the early years of the study period, may have compounded this issue.

Molar progression is also age-dependent [19,20,21,55], with several authors suggesting this is one of the main drivers of the disease [10,21,38,39]. In addition, exposure to soft, low fibre, high sugar artificial diets have been linked to reduced tooth wear and prolonged molar progression [6,10], which can result in oral conditions associated with MPPD, such as “softened oral mucosa”, gingivitis, plaque and calculus [6]. The prolonged exposure to risk factors associated with the development of MPPD, such as an inappropriate diet, coupled with the continuous process of molar progression throughout life, could explain the incremental increase in risk of MPPD that we observed across both regions. This forms the basis for our recommendations to increase oral examinations in older macropods in order to improve early detection and treatment success, and is an important area for future investigation.

The relationship between age and MPPD can be explained by multiple biologically plausible pathways, including decreased immune competency with advanced age [56], longer exposure to environmental risk factors, and likely increased lifespan in captivity [42]. Ageing leads to a number of changes to the dental arcade, such as reduced salivary flow, which could predispose to MPPD [9,19,57]. If earlier stages of disease had been included in the case definition, the association with increasing age would still have been expected, as Kido et al. [3] also reported an association with age.

#### 4.2.3. Genus

MPPD was reported across all genera, suggesting all macropods are likely susceptible to this disease. Species within the *Wallabia, Macropus* and *Petrogale* genera were all at similar risk of disease; with only *Setonix* significantly less likely to have the disease than the other genera. *Setonix* may have a higher resilience to stressors found in captivity, such as visitor presence [58], although notably the majority of this species (27.9%, 85/305) were housed at Zoo A3, which also had a significantly lower burden of disease. Thus, there is a risk that the results for this species are confounded by institution, and future studies of the disease risk in this species would resolve the influence of institution. *Setonix* are browsers and retain premolars, a feature notable in some other macropod species, which subsequently blocks molar progression [8,59,60]; therefore, we may expect to observe a lower risk of MPPD in the browser group. However, this concept is not supported more broadly in our dataset, as we identified similar risks between *Macropus* (grazers) and *Wallabia* (browsers). Although smaller sample sizes at the species level may influence our dataset and ability to detect a true association, it appears institutional and regional management practices may be more likely to influence the incidence of MPPD than the species itself.

#### 4.2.4. Institution

Zoological institutions vary in the species they manage and the practices they use to manage them, and these practices will influence the occurrence of disease. Whilst the IR of MPPD at European institutions did not differ significantly between institutions, in the Australian region one zoo (Zoo A3) had a significantly lower IR, suggesting they may have managed their macropod collection differently from the other Australian institutions in this study. The Poisson regression model established that, compared to the zoo referred to as Zoo A3, the remaining zoos (Zoos A1, A2 and A4), were all at significantly greater risk of developing MPPD. Overall, Zoo A1 housed the largest number of macropods over the study period, and also managed a large proportion of *Petrogale* (70.5% *n* = 643); a genus known to be at risk of MPPD [2,9,61]. In contrast, Zoo A3′s study population was nearly one-third *Setonix* (27.9% *n* = 305), a genus with reported low incidence of MPPD [2]. Therefore, the presence of *Setonix* in greater numbers than the other institutions may have resulted in the relatively low IR for this institution. In addition to housing different genera, the captive management, housing and husbandry of macropods varied between institutions, aspects of management that all have the potential to influence the occurrence of MPPD. As a result, it can be difficult to determine if institutionally based differences reflect the genera/species they house, or the management practices and environment in which those genera/species are found.

Pathways by which institutional management practices and environment can influence the health of macropods, and thus the IR of MPPD, include enclosure size and type [47,58,62], and substrate provided [12]. Overcrowded enclosures have previously been postulated as having an associative role with MPPD, through environmental loading with faecal bacteria, as well as stress [12,18], potentially associated with enclosure type [58]. Although all Australian institutions in our study (when visited for data collection) had walk-through enclosures of varying size, they also exhibited institutional differences in the number of macropod species managed, enclosure contents and substrates. The substrate in particular may play an important role in the development of MPPD; with substrates that hold moisture, such as soil and grass, being more likely to harbour pathogenic bacteria [12]. Environmental exposure to pathogenic bacteria has been reported to increase the risk of morbidity in horses and sheep [63,64], and exposure to bacterial species, including those associated with MPPD (*Fusobacterium necrophorum* [64]), may increase the risk of MPPD in macropods [6,12]. In Australia, the zoo with the lowest IR of MPPD (Zoo A3) housed its macropods on sand, proportionally more so than the other institutions. Sand is a porous substrate, a quality that may be of benefit for reducing the ability of harmful bacteria to be maintained in the ground [64], which could help to explain the lower IR of MPPD for this institution. However, if environmental transmission of pathogenic bacteria were a significant factor in the development of MPPD, then saliva, purulent discharge, and faeces would potentially be the vehicles of such transmission, and climatic conditions would determine how long these matrices remained viable for those bacteria, rather than the substrate.

#### 4.2.5. Stress (Intra- and Inter-Zoo Transfers)

Temporary and permanent intra-zoo transfers of macropods can occur for a variety of reasons including health concerns (movement of an individual to a hospital enclosure), the management of behaviour problems such as agonistic behaviour between males [48], to balance population ratios [47], or to manage captive breeding programs [61]. In addition, zoos are dynamic spaces and may often look for ways to improve enclosures for their animals [42], which may require moving animals either temporarily or permanently to new enclosures within the grounds of the zoo. Our study found that macropods housed in Australian institutions experienced a large number of enclosure transfers (up to 37) during their lifetime, exposing them to the welfare impacts associated with these movements which are likely to be similar to those found in non-macropod species [16,65,66]. The facilitation of pre-transportation examinations may be more likely to be carried out in the Australian zoos due to a greater presence of veterinary personnel. This aspect may increase both the detection and the reported incidence of MPPD for the Australian region, confounding the ability to determine a true association between inter- and intra-zoo transfers and the development of MPPD. Whilst the purpose of some of these moves may be to reduce stress, the movement itself is associated with stressors including pre-transportation procedures often including immobilisation with or without chemical restraint, the transport itself, confinement and adaptation to a new environment [67]. The resulting response may be physiological or behavioural; and fear/flight responses can result in fence-running [8,16,68], which may result in traumatic facial injuries that in macropods can be a precursor for infections, including MPPD [2,68]. Additionally, when MPPD is present in a collection, intra-zoo transfers may also increase the risk of bacterial spread, and contaminate enclosures; yet this process is subject to the influence of climatic conditions on the bacteria released into the environment during transfers. Multiple transfer experiences were correlated with the development of MPPD in Australian institutions, with both inter- and intra-zoo transfers being significantly associated with the disease. Transfers to onsite veterinary facilities, including those for routine health checks, were included as ‘intra-zoo transfers’ in our study, and this could influence the results, as some of these transfers would have been related to the occurrence of the disease itself. However, the inter-zoo transfer relationship is not affected by this confounding factor, and suggests a genuine relationship exists between animal transfers and MPPD. In the Australian region, the vast distances, and subsequent extended duration of travel to other institutions, may increase the risk for, and impact of, transport associated stressors. Notably, the inter- and intra-zoo transfer effect was not significant in the European region. With only half of the European institutions having veterinary facilities onsite, this reduced the potential for macropods in European zoos to be transferred. Further studies could investigate the reasons for inter- and intra-zoo transfers, and the effect of intra-zoo transfers where movements for hospitalisation or medical management are evaluated independently of management movements. Although there may be valid conservation and welfare-related reasons for transferring animals between enclosures and zoos, to reduce the risk of developing MPPD and other welfare impacts, a reduction in overall numbers of transfers, along with a review of biosecurity practices, is recommended. Additionally, to reduce the risk of oral trauma associated with handling and transport, appropriate efforts to train macropods for transportation is recommended. For example, using positive reinforcement techniques [69], by habituating macropods to transfer-related procedures, and/or through providing individuals with positive handling experiences prior to transport [70].

#### 4.2.6. Study Period

Our study found the incidence rate of MPPD was stable across the entire study period for the Australian region, and had increased in the 2005–2016 time period in Europe. This increase may reflect changes in husbandry and management practices for this region; however, the often dynamic nature of these practices can make evaluating these relationships difficult, particularly across institutions. This apparent increase in risk may also be related to an increase in veterinary presence and better recognition of the disease in more recent years, in addition to methodological issues, such as the quality and completeness of records, which varied between regions, institutions and time periods, and included missing and incomplete data. This issue of missing and incomplete data was more common for institutions in Europe than in Australia, and may be the result of varying levels of veterinary support at the institutional level, to facilitate the completion of veterinary records. It is also important to note that healthy macropods in captivity may not always receive veterinary attention, therefore a medical record may never be established for these individuals.

To better understand the relationship between husbandry and management practices, and temporal changes in MPPD incidence rates, would require a detailed study of each institution. Ideally, a prospective study would be used whereby changes are implemented in a stepwise fashion whilst controlling for other risk factors, and cohorts are monitored over reasonable time frames to detect an increase or decrease in the incidence of disease. Given the relatively low IR of MPPD, the chronicity of disease, and numbers of macropods housed in some zoos, it may be difficult to effectively design prospective studies to evaluate specific risk factors related to housing and husbandry. Nonetheless, we emphasise that prospective studies would be particularly beneficial for investigating housing, husbandry and taxonomic variables that may be associated with the development of this disease. Furthermore, we encourage zoos to contribute to the ZIMS database, and recommend its use in future health studies involving zoo animals. To improve the accuracy and increase the power of future research, we recommend that institutions have standardised case definitions and data entry protocols that include thorough, comprehensive details of health events, including MPPD.

### 4.3. Outcome

For captive macropods that develop MPPD, the outcome is frequently death; in this study, 62.5% of the macropods identified as having MPPD eventually succumbed to the disease. Contrary to previous studies, often based on observations of MPPD at necropsy, our results provide an overview of outcomes from MPPD in live populations of captive macropods. The results from this study support previous reports that treatment for MPPD is largely unrewarding [2,34], but this could be due to late detection of the disease and treatment protocols. The proportion of macropod deaths following initial diagnosis for MPPD in the European region (60.4% of diagnosed individuals) may indicate that either treatments are not successful, or that elective euthanasia or unassisted death occurs prior to treatment. Interestingly, clinical resolutions were greater in Europe (24.5% vs. 19.2% in Australia), which may be due to a greater value of individuals, and hence more effort put into treating cases in Europe compared with Australia. These figures also potentially indicate a more efficacious treatment approach in the European region, or improvements in treatment protocols over time in that region. Uncertainties around these hypotheses are related to the small sample sizes for the region. Recurrence of MPPD was expected in both regions, based on previous studies [2,14]; however, recurrence was more commonly observed in the Australian region. All institutions in the Australian region (but only half in the European region) had onsite veterinary services, and this difference may have facilitated treatment, rather than opting to euthanise initial cases of MPPD in Australian institutions. That is, macropods that were treated for MPPD lived, and therefore had a greater chance of a recurrence than if they had not received treatment, thereby contributing to a higher observed rate of recurrence.

The outcome for macropods with MPPD may depend on several factors including the age and health of the individual, the treatment delivered, institutional management considerations influencing treatment versus euthanasia, and potentially the individuals’ genetic value to the collection [42]. Additionally, changes in dental morphology related to generations of captive breeding may result in a ‘genetic susceptibility’ to the disease, as observed in other captive species [35,71,72]. This question is worthy of further investigation, as it would assist zoos with their conservation breeding programs, and may specifically be of benefit with respect to the rarer macropod species housed in captivity. MPPD is frequently fatal, and decisions to euthanise are based on the welfare implications of this chronic and painful disease. The large proportion of macropods that die as a result of the disease emphasises the need for further research into treatment efficacy and preventative measures.

## 5. Conclusions

The aims of this study were to undertake an epidemiological study of MPPD in captive macropods, and identify host and environment-related risk factors associated with development of clinical disease. The risk of developing MPPD was influenced by sex, age, time period and management practices. Specifically increasing age and the number of inter- and intra-zoo transfers had a strong effect on the development of the disease, although regional effects were often identified. Detailed examination at the institutional level is recommended to extract the specific management practices that may have resulted in differences in disease risk between institutions.

The findings from this study show that MPPD remains a significant problem for zoological institutions, and efforts should be made to continue to investigate and clarify risk factors within housing and husbandry systems. Whilst the risk factor analyses provided biologically plausible associations, the hypotheses generated from this study compels the use of robust prospective epidemiological study designs in future research. In addition to the need for further research, we recommend the following to reduce the risk of developing clinical MPPD: (i) increase clinical examinations as macropods age, and (ii) reduce the frequency of inter- and intra-zoo transfers. This new information may assist zoological institutions in reducing MPPD mortality rates and improve the future health and welfare of macropods in captivity.

## Figures and Tables

**Table 1:** Summary of macropods housed at eight zoological institutions across Australia and Europe between 1 January 1995 and 28 November 2016. MPPD = Macropod Progressive Periodontal Disease. For sex, ‘m’ = male, ‘f’ = female, and ‘ud’ = undetermined.

Region	Institution	Total Population	Sex	No. of Genera	No. of Species	Total MPPD Cases
Australia	A1	643	257.359.27	5	11	89
	A2	354	151.190.13	7	17	66
	A3	305	100.194.11	7	15	22
	A4	318	135.165.18	8	19	47
Europe	E1	60	25.34.1	1	1	10
	E2	224	109.112.3	3	8	25
	E3	84	41.35.8	2	5	11
	E4	66	32.28.6	1	1	7

**Table 2:** Incidence rates (IR) (cases/100 animal years) and 95% confidence intervals (CI) for MPPD by sex, age group, study period and institution, for macropods housed at eight Australian and European institutions between 1 January 1995 and 28 November 2016.

Risk Factor	Australia IR (95% CI)	Europe IR (95% CI)
**Sex**		
Male	5.6 (4.3–7.1)	6.2 (4.0–9.2)
Female	5.9 (5.0–7.0)	4.1 (2.7–6.1)
**Age**		
<1	1.9 (1.0–3.2)	2.2 (0.7–5.2)
1–5	5.8 (4.7–7.1)	5.4 (3.4–8.0)
5–9	6.5 (5.0–8.4)	4.4 (2.1–8.1)
10+	12.3 (8.4–17.3)	9.3 (4.6–16.7)
**Study period**		
1995–1999	5.5 (4.1–7.2)	1.1 (0.1–3.9)
2000–2004	6.6 (4.9–8.6)	4.6 (2.0–9.1)
2005–2009	6.8 (5.1–9.0)	6.5 (3.7–10.5)
2010–2016	4.6 (3.4–6.0)	5.8 (3.7–8.7)
**Institution**		
A1/E1	7.6 (6.0–9.4)	5.3 (2.6–9.8)
A2/E2	7.4 (5.7–9.4)	5.0 (3.1–7.4)
A3/E3	2.4 (1.4–3.9)	5.9 (2.8–10.9)
A4/E4	4.5 (3.2–6.0)	3.6 (1.4–7.3)

**Table 3:** Adjusted incidence rate ratios (IRR) for MPPD risk for macropods housed at eight zoological institutions across Australia and Europe between 1 January 1995 and 28 November 2016.

Risk Factor	Adjusted Incidence Rates			
	Australia		Europe	
	IRR (95% CI)	*p*-Value	IRR (95% CI)	*p*-Value
**Study period**				
2000–2004	1.20 (0.82–1.77)	0.345	4.60 (1.14–30.56)	0.055
2005–2009	1.10 (0.74–1.63)	0.633	7.39 (2.06–47.21)	0.008 **
2010–2016	0.75 (0.50–1.11)	0.154	6.94 (1.96–44.18)	0.010 **
**Age group**				
1–4	3.36 (1.95–6.31)	<0.001 ***	2.46 (1.02–7.32)	0.067
5–9	3.92 (2.22–7.47)	<0.001 ***	2.72 (0.94–8.93)	0.075
10+	7.63 (4.06–15.20)	<0.001 ***	7.38 (2.50–24.85)	<0.001 ***
**Institution**				
Zoo A1/E1	3.88 (2.32–6.91)	<0.001 ***	1.38 (0.52–3.86)	0.524
Zoo A2/E2	3.27 (1.94–5.86)	<0.001 ***	1.12 (0.50–2.86)	0.790
Zoo A4/E3	2.09 (1.19–3.84)	0.013 **	1.20 (0.44–3.45)	0.723
**Sex**				
Male	1.08 (0.80–1.44)	0.611	2.02 (1.08–3.83)	0.029 *

Reference categories set as ‘1995–1999′ for calendar period; ‘<1 year’ for age group; ‘females’ for sex; Zoo A3 for Australian institution and Zoo E4 for European institution. * *p* ≤ 0.05, ** *p* ≤ 0.01; *** *p* ≤ 0.001.

**Table 4:** Odds ratios and 95% CI for inter-zoo transfers in relation to risk of developing MPPD in macropods housed in zoological institutions in the Australian and European regions between 1 January 1995 and 28 November 2016. Reference category: macropods that had no inter-zoo transfers.

Number of Inter-Zoo Transfers	Adjusted Incidence Rates			
	Australia		Europe	
	OR (95% CI)	*p*-Value	OR (95% CI)	*p*-Value
0	-	-	-	-
1	1.69 (1.23–2.32)	0.001 ***	0.91 (0.48–1.71)	0.77
2	1.92 (1.19–3.10)	0.01 **	2.4 (0.73–7.89)	0.14
3	3.29 (1.48–7.30)	0.002 **	2.37 (0.10–59.45)	1
4	1.75 (0.20–12.18)	0.48	-	-
5	8.76 (1.22–63.02)	0.06	-	-
6	17.53 (1.57–195.35)	0.03 *	-	-
7	43.60 (2.08–915.60)	0.01 **	-	-

* *p* ≤ 0.05, ** *p* ≤ 0.01, *** *p* ≤ 0.001.

**Table 5:** Odds ratios for intra-zoo transfers in relation to risk of developing MPPD in macropods housed in zoological institutions in the Australian and European regions between 1 January 1995 and 28 November 2016. Reference category: macropods that had no intra-zoo transfers.

No. Intra-Zoo Transfers	Estimates			
	Australia		Europe	
	OR (95% CI)	*p*-Value	OR (95% CI)	*p*-Value
0	-	-	-	-
1	1.58 (0.78–3.22)	0.20	1.67 (0.82–3.39)	0.15
*2*	2.68 (1.36–5.30)	0.003 **	0.83 (0.24–2.88)	1
*3*	4.07 (2.06–8.04)	<0.0001 ***	1.09 (0.06–21.52)	1
*4*	6.61 (3.35–13.05)	<0.0001 ***	2.53 (0.10–63.56)	1
*5*	5.26 (2.45–11.3)	<0.0001 ***	-	-
*6–10*	9.93 (5.57–17.73)	<0.0001 ***	-	-
*11+*	16.18 (8.73–29.98)	<0.0001 ***	-	-

** *p* ≤ 0.01, *** *p* ≤ 0.001.
